# Supplementary material for: A systematic review of the effectiveness and implementation readiness of psychosocial interventions for psychosis in South Asia
Source: PLoS One. 2023 Nov 2;18(11):e0283411. doi: 10.1371/journal.pone.0283411 (PMC10621957; doi:10.1371/journal.pone.0283411)
Supplement: S1 Fig — (DOCX) [file pone.0283411.s002.docx]

**Identification of studies via databases and registers**

Records removed *before screening*:

Duplicate records removed

(n = 281)

Records identified from:

Ovid (MEDLINE; Global Health and PsycInfo) (n= 702)

Web of Science (n= 739) Reference lists (n =1)

Totally (n= 1440)

**Identification**

Records screened

(n = 1161)

Records excluded**

(n = 1045)

Full text articles sought for retrieval

(n = 116)

Articles not retrieved

(n = 15)

**Screening**

Articles excluded:

Non appropriate outcome measured (n = 26)

Non appropriate intervention (n=19)

Non appropriate condition of interest (n =11)

Non appropriate population (n = 1)

Review (n=14)

Protocol (n=3)

Not available in English language (n=1)

Full text articles assessed for eligibility

(n = 101)

Studies included in review

(n = 26)

**Included**
